# Clinicopathological variables influencing overall survival, recurrence and post-recurrence survival in resected stage I non-small-cell lung cancer

**DOI:** 10.1186/s12885-020-6621-1

**Published:** 2020-02-24

**Authors:** Chengdi Wang, Yuxuan Wu, Jun Shao, Dan Liu, Weimin Li

**Affiliations:** Department of Respiratory and Critical Care Medicine, West China Hospital, West China Medical School, Sichuan University, No. 37 Guo Xue Alley, Chengdu, 610041 Sichuan China

**Keywords:** Non-small-cell lung cancer, Survival, Recurrence, Risk factors, Post-recurrence survival

## Abstract

**Background:**

To investigate clinicopathological variables influencing overall survival, overall recurrence, and post-recurrence survival (PRS) in patients who experienced curative-intent surgical resection of stage I non-small-cell lung cancer (NSCLC).

**Methods:**

We investigated a series of 1387 patients with stage I NSCLC who underwent surgical resection from 2008 to 2015. The effect clinicopathological factors on death, recurrence, and PRS were evaluated by Kaplan-Meier estimates and cox regression analysis.

**Results:**

Among the 1387 stage I patients, 301 (21.7%) experienced recurrence. The 5-year cumulative incidence of recurrence (CIR) for all patients was 20.2% and median PRS was 25.5 months. The older age (*P* = 0.036), p-stage IB (*P* = 0.001), sublobar resection(*P*<0.001), histology subtype (*P*<0.001), and lymphovascular invasion (LVI) (*P* = 0.042) were significantly associated with worse overall survival. Among 301 recurrent patients, univariable analysis indicated that p-stage IB (versus IA) (*P*<0.001), LVI (*P*<0.001) and visceral pleural invasion (VPI) (*P*<0.001) were remarkably correlated with the higher incidence of recurrence. Taking the effect of clinicopathological variables on PRS into consideration, smoking history (*P* = 0.043), non-adenocarcinoma (*P* = 0.013), high architectural grade of LUAD (*P* = 0.019), EGFR wild status (*P* = 0.002), bone metastasis (*P* =0.040) and brain metastasis (*P* = 0.042) were substantially related with poorer PRS. Multivariate analysis demonstrated that high architectural grade of LUAD (*P* = 0.008), brain metastasis (*P* = 0.010) and bone metastasis (*P* = 0.043) were independently associated with PRS.

**Conclusion:**

In patients with resected stage I NSCLC, the older age, p-stage IB (versus IA), sublobar resection, histology subtype, and LVI were significantly associated with worse overall survival. P-stage IB (versus IA), LVI, and VPI were significantly correlated with the higher incidence of recurrence. High architectural grade of LUAD, brain metastasis and bone metastasis were independent risk factors with PRS.

## Background

Lung cancer is so far the leading cause of cancer-related mortality, accounting for an estimate of 690, 000 deaths in China and 1,761,000 deaths worldwide in 2018 [1, 2]. The standard of care for patients with early-stage non-small-cell lung cancer (NSCLC) is the curative-intent anatomic surgical resection, whereas tumor metastasis or recurrence leads to the treatment failure and mortality after surgery [3]. Reported locoregional recurrence rates were shown to elevate with advancing pathological stage (5–19%, 11–27%, 24–40% for stage I, II, and IIIA respectively) and to range with various surgical resection modalities (lobectomy, 4.9–7%; segmentectomy, 9.1–16%; and wedge resection, 11–27.8%) [4]. Previous studies have reported that recurrence rates, based on the primary stage and follow-up time, varied between 18.5 and 75% for resected NSCLC patients with stage I to III [5–7]. According to outcomes of the National Lung Screening Trial (NLST) and the Nelson trials for screening computed tomography (CT) scans, the improvements in the early diagnosis and the reduction in the mortality of lung cancer have been greatly anticipated [8, 9]. Appropriate surveillance strategies such as CT scans are therefore of great importance to identify earlier and to screen recurrent patients who have the high probability of mortality. Hence, identification of prognostic variables for recurrence in lung cancer after surgery is of great significance for screening high-risk patients for further and better treatments.

NSCLC accounts for approximately 85% of lung cancer, including the primary subtypes such as lung adenocarcinoma (LUAD), squamous carcinoma (LUSC), and adenosquamous carcinoma (LASC) [10]. LUAD is the most common histologic type of NSCLC, which, based on the predominant subtype, is classified into adenocarcinoma in situ (AIS), minimally invasive adenocarcinoma (MIA), lepidic-, acinar-, papillary-, micropapillary-, and solid-predominant invasive adenocarcinoma (IA) in accordance with IASLC/ATS/ERS and 2015 WHO classifications [11, 12]. Previous studies have demonstrated that the predominant histologic patterns were strongly associated with recurrence-free survival (RFS) [13, 14]. Up to date, several studies have reported the prognostic value of the new classification to predict mortality and recurrence mainly in LUAD or non-LUAD. Nevertheless, few studies were found to focus on LUSC, LASC or other lung cancer subtypes [15–17]. Among these limited number of studies, even fewer evaluated the predictive value of such classification with regard to recurrence patterns and post-recurrence survival (PRS) in NSCLC, especially LUSC [5, 6, 18–20]. To mend this inadequacy, we set out to investigate the prognostic value of clinicopathologic factors and histologic subtypes on the overall survival, overall recurrence, and PRS. Our study involved a large and homogenous cohort of resected stage-I patients with NSCLC, not limited to lung adenocarcinoma or squamous cell carcinoma. By focusing on recurrent patients following the curative-intent surgery, we could identify the risk factors and explore their effect on the OS, overall recurrence, and PRS in resected stage-I NSCLC patients.

## Methods

In this study, we retrospectively reviewed the medical records of all patients who had undergone anatomic resection for pathologically diagnosed stage I NSCLC including LUAD, LUSC, LASC and other histologic subtypes. The medical clinicopathologic data were taken from West China Hospital (WCH), Sichuan University between 2009 through 2015. Lobectomy was deemed to be as the standard surgical modality for early-stage NSCLC patients at WCH. Sublobar resection, including segmentectomy or wedge resection, was regarded as the surgical option for patients with comorbidities, poor pulmonary function, or very small nodules that made lobectomy inappropriate. The clinicopathologic variables were retrieved from our prospectively established Lung Cancer Database of West China Hospital as follows: age (operation age and recurrence age), sex, smoking history, surgery modality, tumor histology, pathologic TNM stage, lymphovascular invasion (LVI), visceral pleural invasion (VPI), EGFR status, adjuvant therapy, PRS. Exclusion criteria were patients who had received preoperative chemotherapy, or/and radiation therapy, or had multiple metachronous or metastatic lesions, or had positive surgical margin. A total of 1387 patients who had the complete follow-up were eligible for the study.

Postoperative assessment contained health checkup, serum tumor markers (CEA, CA125, CA199, NSE, CYFRA21-1), chest/upper-abdominal CT scans, and bone scintigraphy. Histologic subtypes of NSCLC were identified according to the IASLC/ATS/ERS and 2015 WHO classifications. LUAD was classified into MIA and IA, the latter of which was subdivided into solid-, micropapillary-, papillary-, acinar-, and lepidic-predominant subtypes [11, 12]. Tumors were divided into 3 groups including high grade group of micropapillary- and solid-predominant IA, intermediate group of acinar- and papillary-predominant IA, low group of MIA and lepidic-predominant IA [13, 21]. Disease stage was determined in accordance with the 8th edition of the American Joint Committee (AJCC) on Cancer Staging Manual [22]. The following factors were also included in this study: pathologic stage, visceral pleural invasion (VPI), lymphovascular invasion (LVI), and EGFR status. Routine follow-up of postoperative lung cancer was carried out on the basis of National Comprehensive Cancer Network (NCCN) guidelines [23]. Medical examination, blood examination (serum tumor biomarkers), chest or and abdomen CT scans were performed every 6 months for the first 2 years after resection. The clinical follow-up and routine CT scans were carried out annually from the 3rd to 5th year after surgery. Brain magnetic resonance imaging (MRI), abdominal and cervical/supraclavicular ultrasonography, or bone scintigraphy were done if abnormal symptoms were noticed in the corresponding regions. All the data were extracted from the Lung Cancer Database of West China Hospital, which covered the clinicopathological characteristics and complete follow-up information of included patients. The current study was approved by the Institutional Review Board of West China Hospital, Sichuan University, and informed consent was waived by the board because of its retrospective nature.

This study had two main endpoints: (1) recurrence after initial surgery and (2) death with or without recurrence. The identification of recurrence was determined by using the imageological examination such as CT, PET/CT, MRI or obtaining the histological specimen when necessary. Second independent primary lung cancer was distinguished from recurrent or metastatic foci via histologic profile of available biopsy specimen or image omics in accordance with the proposed criteria of the IASLC Lung Cancer Staging Project [24]. Local recurrence was regarded as second loci in the ipsilateral containing the ipsilateral hilus and ipsilateral mediastinum. Distant metastasis or recurrence was deemed as the new lesion in the opposite lung, or elsewhere outside the mediastinum and hemithorax [5].

To investigate the prognostic value of clinicopathologic variables in the OS and overall recurrence, we adopted both univariable and multivariable analyses. The length of OS was calculated between the initial operate date and the time of either death or last contact. The length of overall recurrence was measured from the date of resection to the time of initial recurrence. Length of PRS was deemed as the interval between the initial recurrence date and death date or last contact. Patients were censored at the last available follow-up when they had not experienced death or relapse. We performed the Kaplan-Meier approach on the basis of log-rank test to estimate the OS and PRS. Cumulative incidence of recurrence (CIR) was calculated by adopting the probability of recurrence after surgery based on competing risks approaches [25]. We performed the Gray method for univariable nonparametric tests and used Fine-Gray model for multivariable analyses to assess the differences in CIR between groups [26, 27]. SPSS software (version 21.0) and R version 3.6.0 were used to perform the statistical analyses, and two-sided *P* values < 0.05 were regarded as the statistical significance.

## Results

**Fig. 1:**
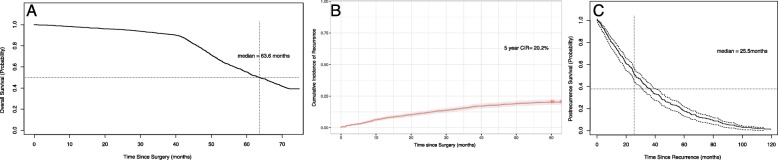
**a** Overall survival of patients with stage I NSCLC; **b** Cumulative incidence of recurrence (CIR) of patients with stage I NSCLC; **c** Post-recurrence survival (PRS) curve for recurrent patients with stage I NSCLC

This study cohort consisted of 1387 patients with resected stage I NSCLC, who met the inclusion and exclusion criteria. Among them were 1028 LUAD including 12 MIA, 276 LUSC, 49 LASC, and 34 other tumor histology subtypes (Others). In the current study, no recurrent disease was observed in AIS or in MIA. Of the 1028 LUAD, 447 patients who had the available subtypes were classified as lepidic predominant (*n* = 183), acinar predominant (*n* = 178), papillary predominant (*n* = 48), micropapillary predominant (*n* = 2), and solid predominant (*n* = 24). Detailed clinicopathologic characteristics are delineated in Table 1. The median overall survival was more than 60 months and the median follow-up for the identified 1387 patients with NSCLC was 63.6 months (range: 61.6–65.5 months) (Fig. 1a). At the end of the study period, 251 patients had died. The older age (HR: 1.169, 95%CI: 1.010–1.352; *P* = 0.036), p-stage IB (HR: 1.217, 95%CI: 1.106–1.461; *P* = 0.001), sublobar resection (HR: 1.548, 95%CI: 1.280–1.871; *P*<0.001) and histologic subtype (*P*<0.001), and lymphovascular invasion (LVI) (*P* = 0.042) were significantly associated with overall survival.

**Table 1:** Patient characteristics and univariable analysis of overall survival and overall recurrence

		Overall Survival (*n* = 1387)				Overall Recurrence				
		Univariate Analysis		Multivariate Analysis		Univariate Analysis			Multivariate Analysis	
	N	HR 95%CI	*P* value	HR 95%CI	*P* value	5-yr CIR	SHR 95% CI	*P* value	SHR 95% CI	*P* value
Primary tumor factor										
Age at surgery, years										
≤ 65	971	1		1		19.9%	1			
>65	416	1.169(1.010–1.352)	0.036	1.112(0.898–1.376)	0.330	21.0%	1.063(0.826–1.368)	0.633		
Sex										
Male	772	1				21.7%	1			
Female	615	0.965(0.930–1.220)	0.364			18.3%	0.821(0.647–1.041)	0.104		
Smoking history										
Never	783	1		1		18.9%	1			
Ever	604	0.875(0.762–1.004)	0.057	1.152(1.026–1.432)	0.043	21.9%	1.192(0.944–1.506)	0.105		
Pathologic stage										
IA	488	1		1		12.8%	1		1	
IB	899	1.217(1.106–1.461)	0.001	1.318(1.071–1.621)	0.010	24.2%	2.048(1.547–2.710)	< 0.001	1.123(0.633–1.994)	0.692
Surgery										
Lobectomy	1223	1		1		20.6%	1			
Sublobar	164	1.548(1.280–1.871)	< 0.001	1.196(0.914–1.564)	0.192	20.7%	1.053(0.590–1.274)	0.468		
Tumor histology										
LUAD	1028	1				19.1%	1			
LUSC	276	0.693(0.576–0.835)				22.3%	1.198(0.901–1.593)			
LASC	49	0.775(0.520–1.155)				30.6%	1.757(1.040–2.970)			
Others	34	1.081(0.700–1.669)	0.001			20.6%	1.115(0.525–2.369)	0.145		
Carcinoma type										
LUAD	1028	1		1		19.1%	1		1	
Non-Non-LUAD	359	0.735(0.623–0.867)	< 0.001	1.041(0.140–1.733)	0.929	23.3%	1.262(0.978–1.629)	0.074	1.987(0.837–2.344)	0.073
Predominant subtype of LUAD										
MIA	12	1		1		8.3%	1		1	
Lepidic	183	0.580(0.322–0.994)		1.446(0.587–3.562)		10.9%	1.293(0.174–9.636)		0.961(0.127–1.261)	
Acinar	178	1.084(0.603–1.950)		1.119(0.615–2.035)		20.7%	2.603(0.357–8.974)		1.833(0.247–3.623)	
Papillary	48	0.877(0.464–1.659)		1.487(0.574–3.856)		25.0%	3.178(0.413–4.443)		1.984(0.251–5.702)	
Micropapillary	2	0.478(0.107–2.137)		0.807(0.800–6.262)		50.0%	10.576(0.661–16.154)		9.424(0.559–10.928)	
Solid	24	1.501(0.746–3.023)	< 0.001	1.611(0.786–3.300)	< 0.001	33.4%	4.911(0.614–9.268)	0.070	2.979(0.368–4.104)	0.030
EGFR status										
Wild-type	206	1				33.0%	1			
Mutated	277	1.032(0.849–1.255)	0.753			27.0%	0.789(0.568–1.095)	0.157		
LVI										
Absent	1336	1		1		19.0%	1		1	
Present	51	1.414(1.013–1.975)	0.042	1.086(0.601–1.996)	0.790	51.0%	3.364(2.247–5.038)	< 0.001	1.586(1.339–2.936)	0.037
VPI										
Absent	818	1				15.9%	1		1	
Present	569	0.899(0.783–1.033)	0.132			26.4%	1.779(1.408–2.248)	< 0.001	1.217(1.073–1.833)	0.006
Adjuvant chemotherapy (stage IB)	899									
No Chemotherapy	555	1				13.5%	1		1	
Chemotherapy	344	1.038(0.870–1.238)	0.678			41.3%	3.925(2.952–5.219)	< 0.001	4.433(2.736–7.813)	< 0.001

*Abbreviations*: *CIR* cumulative incidence of recurrence, *AIS* adenocarcinoma in situ, *MIA* minimally invasive adenocarcinoma, *LVI* lymphovascular invasion, *VPI* visceral pleural invasion, *LUAD* lung adenocarcinoma, *LUSC* lung squamous carcinoma, *LASC* lung adenosquamous carcinoma, *NSCLC* non-small-cell lung cancer

Of the 1387 patients identified, 301 (21.7%) had developed recurrence or relapse. The 5-year overall recurrence for all stage I patients was 20.2% (Fig. 1b). Table 1 presented results of univariate and multivariate analyses of overall survival and overall recurrence according to clinicopathologic characteristics of patients with stage I NSCLC. For univariate analysis, p-stage IB (versus IA) (HR: 2.048, 95%CI: 1.547–2.710; *P*<0.001), LVI (HR: 3.364, 95%CI: 2.247–5.038; *P*<0.001), visceral pleural invasion (VPI) (HR: 1.779, 95%CI: 1.408–2.248; *P*<0.001) were significantly correlated with the higher incidence of lung cancer recurrence.

**Fig. 2:**
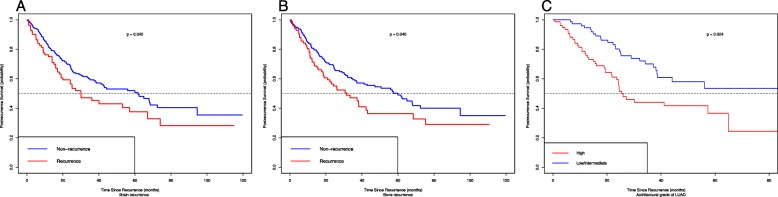
Post-recurrence survival (PRS) curve for recurrent patients with stage I NSCLC by subgroups into brain recurrence status (**a**), bone recurrence status (**b**), architectural grade of LUAD (**c**)

Of the 301 patients who underwent the recurrence, 230 (76.4%) had distant recurrence, 71 (23.6%) had local recurrence, and 141 died during the at least 5-year follow-up. The most commonly involved organs for distant recurrence were the lung (*n* = 193), brain (*n* = 82), bone (*n* = 85) and liver (*n* = 30). The majority of recurrences were diagnosed by CT scans. A total of 194 recurrent patients received the post-recurrence therapy (PRT), including chemotherapy for 67 patients, surgery plus chemotherapy or and targeted therapy for 34, targeted therapy alone for 22, surgery alone for 3 (Table 2). Other treatments details are presented in Table 2. On the whole, 1-,2- and 5-year PRS was 75.1%, 55.1, and 16.6% respectively. Median PRS time for the recurrent patients was 25.5 months (range: 22.2–28.9 months) (Fig. 1c). We further explored risk factors associated with post-recurrence survival. Taking the effect of clinicopathological variables on PRS into the account, smoking history (HR:1.266, 95%CI: 1.008–1.589; *P* = 0.043), non-adenocarcinoma (HR: 1.357, 95%CI: 1.074–1.762; *P* = 0.013), high architectural grade of LUAD (HR: 2.795, 95%CI:1.181–6.615; *P* = 0.019), EGFR wild status (HR:2.140, 95%CI: 1.307–3.503; *P* = 0.002), brain metastasis (HR: 1.442, 95%CI:1.013–2.051; *P* = 0.042) and bone metastasis (HR: 1.443, 95%CI:1.017–2.048; *P* = 0.040) were significantly related with worse PRS (Fig. 2). Multivariate analysis revealed that high architectural grade of LUAD (HR: 3.740, 95%CI:1.405–9.953; *P* = 0.008), brain metastasis (HR: 3.557, 95%CI:1.354–9.340; *P* = 0.010) and bone metastasis (HR: 2.397, 95%CI:1.026–5.601; *P* = 0.043) were independently and significantly associated with PRS.

**Table 2:** Patient characteristics and PRS analysis

Overall	Univariate Analysis			Multivariate Analysis	
Recurrent Patients	No. (%)	HR (95% CI)	*P* value	HR (95% CI)	*P* value
Primary tumor factor	301				
Age at recurrence, years					
≤ 65	195	1			
>65	106	1.187(0.936–1.506)	0.157		
Sex					
Male	178	1			
Female	123	0.861(0.683–1.085)	0.204		
Smoking history					
Never	163	1		1	
Ever	138	1.266(1.008–1.589)	0.043	1.847(0.541–6.313)	0.328
Pathologic stage					
IA	70	1			
IB	231	1.113(0.718–1.725)	0.633		
Surgery					
Lobectomy	284	1			
Sublobar resection	17	1.183(0.724–1.933)	0.502		
Tumor histology					
Adenocarcinoma (LUAD)	210	1			
Squamous carcinoma (LUSC)	65	1.344(1.016–1.778)			
Adenosquamous carcinoma (LASC)	19	1.319(0.823–2.113)			
Others	7	1.889(0.886–4.025)	0.068		
Carcinoma type					
Adenocarcinoma (LUAD)	210	1		1	
Non-Adenocarcinoma (Non-LUAD)	91	1.375(1.074–1.762)	0.013	7.421(0.861–8.323)	0.909
Architectural grade of LUAD					
Low/immediate grade	76	1		1	
High grade	9	2.795(1.181–6.615)	0.019	3.740(1.405–9.953)	0.008
EGFR status					
Mutated	80	1		1	
Wild-type	77	2.140(1.307–3.503)	0.002	0.385(0.115–1.284)	0.120
Lymphovascular invasion (LVI)					
Absent	284	1			
Present	17	0.749(0.451–1.245)	0.749		
Visceral pleural invasion (VPI)					
Absent	115	1			
Present	186	1.068(0.729–1.566)	0.735		
Type of recurrence					
Local	71	1			
Distant	230	1.009(0.772–1.318)	0.949		
Recurrence pattern					
Intrathoracic	62	1			
Extrathoracic	87	0.756(0.543–1.053)			
Both	152	0.762(0.566–1.027)	0.165		
Recurrence pattern					
Single site	137	1			
Multiple site	164	1.004(0.728–1.148)	0.439		
Recurrence site					
Lung	193	1.198(0.837–1.715)	0.324		
Brain	82	1.442(1.013–2.051)	0.042	3.557(1.354–9.340)	0.010
Bone	85	1.443(1.017–2.048)	0.040	2.397(1.026–5.601)	0.043
Liver	30	1.139(0.685–1.893)	0.617		
Initial therapy of recurrence					
Single therapy					
Surgery	3	0.746(0.239–2.331)	0.614		
Chemotherapy	67	0.896(0.681–1.179)	0.432		
Radiation therapy	20	1.041(0.660–1.640)	0.863		
Targeted therapy	22	0.998(0.891–2.380)	0.095		
Multimodality					
Chemotherapy+ radiation therapy/ targeted therapy	48	0.821(0.602–1.120)	0.213		
Surgery + Chemotherapy/radiation therapy/targeted therapy	34	0.758(0.530–0.984)	0.046	0.663(0.174–2.533)	0.548

*Abbreviations*: *LVI* lymphovascular invasion, *VPI* visceral pleural invasion, *LUAD* lung adenocarcinoma, *LUSC* lung squamous carcinoma, *LASC* lung adenosquamous carcinoma

## Discussion

Although previous studies have reported molecular and clinicopathologic variables for the recurrence for NSCLC after initial resection especially in LUAD [28, 29], the recurrence pattern of LUSC, LASC or other NSCLC subtypes still needs to be investigated. To our knowledge, this present study is the first to comprehensively explore the influence of clinicopathologic factors on OS, overall recurrence and post-recurrence survival based on a largest cohort of patients with NSCLC having LUAD, LUSC, LASC and other subtypes. The median follow-up period of all resected lung cancer patients was more than 60 months.

The prognostic value of the new IASLC/ATS/ERS classification system in the OS and the overall recurrence has been reported and discussed in several previous studies [15, 16, 21, 30]. Warth et al. reported that solid-, micropapillary-, and papillary-adenocarcinoma patients who underwent the surgery (the frequencies: 37.6, 6.8, and 4.7% respectively), compared to lepidic- and acinar-predominant histologic patterns (the frequencies: 8.1 and 42.5%, respectively), were significantly related with lower disease-free survival (DFS) and poorer OS [15]. Yoshizawa et al. showed that LUAD patients with stage I having high-grade tumors including solid- and micropapillary-predominant subtypes were significantly associated with worse overall survival and a higher incidence of recurrence [21]. Hung et al. demonstrated that LUAD patients with resected stage I-III owing the high architectural grade including solid- (13.6%) and micropapillary- (19.5%) predominant patterns, compared with papillary- (27.1%), acinar- (33.7%), and lepidic- (6.1%) predominant subtypes, were remarkably associated with worse overall survival, poorer disease- specific survival and higher incidence of recurrence [16, 31]. Our outcomes also demonstrated that the solid-predominant patients of LUAD had the higher possibility of recurrence similarly to the reported results despite the limited number of corresponding patients. According to the regular CT surveillance protocol, we found that most recurrences or disease progression appeared within the first 2 years after the curative-intent surgical section, which indicated that the regular CT surveillance was of great significance for the postoperative lung cancer patients. However, the best interval time for postoperative follow-up is still to be warranted to be investigated and validated in case of excessive or delayed medical treatment due to insufficient diagnosis. In addition, the current study also demonstrated that high architectural grade including solid-predominant LUAD was significantly associated with poor PRS, which highlights the need for medical care for the postoperative clinical contact.

The present study also investigated the clinicopathological factors influencing the PRS of stage I NSCLC patients. Although surgical resection with curative intent is the most effective treatment modality for patients having stage I NSCLC, previous studies have reported an incidence of recurrence in stage I NSCLC ranging from 14 to 36%, with 1- and 2-year PRS rates of 38–88%, and 19–72.3% respectively (Table 3). In this study, overall incidence of recurrence during the postoperative 5 years was 20.2% and median PRS time was 25.5 months. We examined the impact of clinicopathological variables on OS and overall recurrence and identified a number of risk factors that were significantly associated with worse OS including the older age (*P* = 0.036), p-stage IB (*P* = 0.001), sublobar resection(*P*<0.001), histologic subtype (*P*<0.001), and lymphovascular invasion (LVI) (*P* = 0.042). Smoking history (*P* = 0.043), non-adenocarcinoma (*P* = 0.013), high architectural grade of LUAD (*P* = 0.019), EGFR wild status (*P* = 0.002), bone metastasis (*P* = 0.040) and brain metastasis (*P* = 0.042) were marginally correlated with worse PRS. Some risk factors such as sublobar resection and high architectural grade of LUAD were consistent with previous studies.

**Table 3:** Post-recurrence survival of patients with stage I NSCLC in previous studies

Series	Year	No. of patients	Histologic profile	Recurrence	Incidence of Recurrence (%)	PRS, % (y)	Independent factors of poor PRS
Current study	2019	1387	LUAD: 1028 LUSC: 276 LASC: 49 Others: 34	LUAD:210 LUSC: 65 LASC: 19 Others: 7	301 (21.7%) Locoregional recurrence: 71 (23.6%); Distant metastasis: 230 (76.4%)	75.1% (1-year) 55.1%(2-year) 37.2%(3-year) 16.6%(5-year)	architectural grade (micropapillary and solid predominant); recurrence site of brain or bone
Ujiie et al. [5]	2014	1120	LUAD: 1120	LUAD: 188	188 (17%) Locoregional recurrence: 59 (31%) Distant metastasis: 129 (69%)	67% (1-year) 45% (2-year) 36% (3-year) 14% (5-year)	Older age (>65 yr) at the time of recurrence; sublobar resection; solid predominant; distant metastasis;
Shimada et al. [6]	2013	919	LUAD: 919	LUAD: 46 Non-LUAD: 46	170 (18%) Locoregional recurrence: 43 (25%) distant metastasis: 113 (66%) locoregional recurrence + distant metastasis: 14 (9%)	73% (1-year) 51% (2-year)	PRT; male sex; poorly differentiated
Hung et al. [16]	2013	283	LUAD: 283	LUAD: 283	57 (20%)	72.3% (2-year) 31.6% (5-year)	Micropapillary and solid predominant
Song et al. [20]	2013	475	NSCLC	LUAD: 46 LUSC: 15 Other: 11	72 (15%) Locoregional recurrence: 36 (50%) distant metastasis: 36 (50%)	88% (1-year) 53% (3-year)	Bad response for treatment; Recurrence-free interval<12 months
Hung et al. [7]	2010	933	NSCLC	LUAD: 95 LUSC: 46 Other: 25	Distant metastasis: 166 (17.8%) Single organ metastasis: 106 Multiple organ metastasis: 60	37.7% (1-year) 18.9% (2-year)	Disease-free interval more than 16 months
Hung et al. [19]	2009	933	NSCLC	LUAD: 45 LUSC: 60 Other: 18	Locoregional recurrence: 123 (13.2%) Local only: 74 locoregional recurrence + distant metastasis: 49	48.0% (1-year) 18.7% (2-year)	PRT (chemotherapy, surgery, and/or radiotherapy)
Nakagawa et al. [18]	2008	397	LUAD:300 LUSC: 89 Other: 8	87	87 (21.9%) Locoregional recurrence: 30 (34.5%) Distant metastasis: 57 (65.6%)	67.7% (1-year) 34.4% (3-year)	Symptoms at recurrence: liver or cervico-mediastinum; PRT (non-surgery/surgery)

*Abbreviations*: *LASC* lung adenosquamous carcinoma, *LUAD* lung adenocarcinoma, *LUSC* lung squamous carcinoma, *PRT* post-recurrence therapy

Previous research reported that the recurrence sites might be a risk factors for PRS, which was consistent with our findings. Yoshino et al. showed that bone metastasis was reported to be the remarkably significant unfavorable factor for PRS in the NSCLC patients with resected stage I-III [32]. Shimada et al. demonstrated that liver metastasis (*P*<0.001) and bone metastasis (*P* = 0.001) were independently and significantly correlated with worse PRS [6]. Ujiie et al. showed that solid predominant adenocarcinoma was marginally associated with higher recurrence or metastasis incidence of brain (*P* = 0.007), adrenal gland (*P* = 0.034), and liver (*P* = 0.038) than the non-solid predominant tumors [5]. Hung et al. reported that the higher incidence of distant metastasis occurred in adenocarcinoma and higher probability of local recurrence existed in non-adenocarcinoma [33]. Zhang et al. confirmed that adenocarcinoma histology, compared to squamous cell carcinoma, had the higher incidence of bone or brain recurrence [34]. The present study also indicated that the non-LUAD histology, brain metastasis and bone metastasis were significantly associated with worse PRS.

With the rapid development of management of lung cancer, molecular target therapy of tyrosine kinase inhibitors (TKI) has exerted survival benefit for the NSCLC patients with EGFR mutations [35, 36]. Shimada et al. demonstrated that epidermal growth factor receptor-tyrosine kinase inhibitors (EGFR-TKIs), compared with platinum-based doublet chemotherapy, were significantly associated with favorable PRS (HR = 0.460, 95%CI 0.245–0.862, *P* = 0.015), which improved the quality of life and survival benefit [6]. The current study also suggested that NSCLC patients with EGFR mutations, having received the EGFR-TKIs, obtained a favorable PRS. However, since no EGFR mutations accounts for the majority of the lung cancer, the most appropriate treatment modality for resected lung cancer with no mutations is needed to be investigated.

Nonetheless, the present study had some limitations. First, the retrospective nature hinders us to assess the influence of clinicopathological factors on the post-recurrence survival. Prospective randomized controlled trials (RCTs) are more appropriate in this regard. Second, our sample may not be largely representative because all patients involved in the study were Chinese. A multi-center investigating targeting non-Asian populations will certainly validate the results. Finally, not all LUADs had the predominant histologic subtypes due to insufficient records data. Despite these limitations, this current study is, to our knowledge, the first to investigate comprehensively the impact of clinicopathologic factors on post-recurrence survival based on the largest cohort of patients diagnosed with NSCLC with a median follow up of more than 5 years.

## Conclusion

In conclusion, the clinicopathological variables have significant prognostic and predictive value for the OS, overall recurrence, and PRS, which will likely affect the clinical decision making in the near future. This study also provides new insight to help clinicians to identify high-risk patients, make personalized postoperative follow-up strategies and conduct the appropriate post-recurrence therapies.

## Data Availability

The original data that support the results of this study are available from the corresponding authors upon reasonable request.

## Abbreviations

AIS: Adenocarcinoma in situ
CIR: Cumulative incidence of recurrence
LASC: Lung adenosquamous carcinoma
LUAD: Lung adenocarcinoma
LUSC: Lung squamous carcinoma
LVI: Lymphovascular invasion
MIA: Minimally invasive adenocarcinoma
NSCLC: Non-small–cell lung cancer
PRT: Post-recurrence therapy
RCTs: Randomized controlled trials
VPI: Visceral pleural invasion

## References

[1] Bray F, Ferlay J, Soerjomataram I (2018). Global cancer statistics 2018: GLOBOCAN estimates of incidence and mortality worldwide for 36 cancers in 185 countries. CA Cancer J Clin.

[2] Davies MPA, Cheng YI, Field JK (2019). Implementation planning for lung cancer screening in China. Pre Clin Med.

[3] Martini N, Bains MS, Burt ME (1995). Incidence of local recurrence and second primary tumors in resected stage I lung cancer. J Thorac Cardiovasc Surg.

[4] Mahvi DA, Liu R, Grinstaff MW (2018). Local Cancer recurrence: the realities, challenges, and opportunities for new therapies. CA Cancer J Clin.

[5] Ujiie H, Kadota K, Chaft JE (2015). Solid predominant histologic subtype in resected stage I lung adenocarcinoma is an independent predictor of early, extrathoracic, multisite recurrence and of poor postrecurrence survival. J Clin Oncol.

[6] Shimada Y, Saji H, Yoshida K (2013). Prognostic factors and the significance of treatment after recurrence in completely resected stage I non-small cell lung cancer. Chest.

[7] Hung JJ, Jeng WJ, Hsu WH (2010). Prognostic factors of postrecurrence survival in completely resected stage I non-small cell lung cancer with distant metastasis. Thorax.

[8] Aberle DR, Adams AM, Berg CD (2011). Reduced lung-cancer mortality with low-dose computed tomographic screening. N Engl J Med.

[9] De Koning H, Van Der Aalst C, Ten Haaf K (2018). PL02.05 effects of volume CT lung cancer screening: mortality results of the NELSON randomised-controlled population based trial. J Thorac Oncol.

[10] Herbst RS, Morgensztern D, Boshoff C (2018). The biology and management of non-small cell lung cancer. Nature.

[11] Travis WD, Brambilla E, Noguchi M (2011). International Association for the Study of Lung Cancer/American Thoracic Society/European Respiratory Society international multidisciplinary classification of lung adenocarcinoma. J Thorac Oncol.

[12] Travis WD, Brambilla E, Nicholson AG (2015). The 2015 World Health Organization classification of lung tumors: impact of genetic, clinical and radiologic advances since the 2004 classification. J Thorac Oncol.

[13] Sica G, Yoshizawa A, Sima CS (2010). A grading system of lung adenocarcinomas based on histologic pattern is predictive of disease recurrence in stage I tumors. Am J Surg Pathol.

[14] Kadota K, Suzuki K, Kachala SS (2012). A grading system combining architectural features and mitotic count predicts recurrence in stage I lung adenocarcinoma. Mod Pathol.

[15] Warth A, Muley T, Meister M (2012). The novel histologic International Association for the Study of Lung Cancer/American Thoracic Society/European Respiratory Society classification system of lung adenocarcinoma is a stage-independent predictor of survival. J Clin Oncol.

[16] Hung JJ, Jeng WJ, Chou TY (2013). Prognostic value of the new International Association for the Study of Lung Cancer/American Thoracic Society/European Respiratory Society lung adenocarcinoma classification on death and recurrence in completely resected stage I lung adenocarcinoma. Ann Surg.

[17] Russell PA, Wainer Z, Wright GM (2011). Does lung adenocarcinoma subtype predict patient survival?: a clinicopathologic study based on the new International Association for the Study of Lung Cancer/American Thoracic Society/European Respiratory Society international multidisciplinary lung adenocarcinoma classification. J Thorac Oncol.

[18] Nakagawa T, Okumura N, Ohata K (2008). Postrecurrence survival in patients with stage I non-small cell lung cancer. Eur J Cardiothorac Surg.

[19] Hung JJ, Hsu WH, Hsieh CC (2009). Post-recurrence survival in completely resected stage I non-small cell lung cancer with local recurrence. Thorax.

[20] Song IH, Yeom SW, Heo S (2014). Prognostic factors for post-recurrence survival in patients with completely resected stage I non-small-cell lung cancer. Eur J Cardiothorac Surg.

[21] Yoshizawa A, Motoi N, Riely GJ (2011). Impact of proposed IASLC/ATS/ERS classification of lung adenocarcinoma: prognostic subgroups and implications for further revision of staging based on analysis of 514 stage I cases. Mod Pathol.

[22] Amin MBCancer (2016). AJCo. AJCC Cancer staging manual.

[23] Rubins J, Unger M, Colice GL (2007). Follow-up and surveillance of the lung cancer patient following curative intent therapy. Chest.

[24] Detterbeck FC, Franklin WA, Nicholson AG (2016). The IASLC lung Cancer staging project: background data and proposed criteria to distinguish separate primary lung cancers from metastatic foci in patients with two lung tumors in the forthcoming eighth edition of the TNM classification for lung cancer. J Thorac Oncol.

[25] Dignam JJ, Zhang Q, Kocherginsky M (2012). The use and interpretation of competing risks regression models. Clin Cancer Res.

[26] Fine JP, Gray RJ (1999). A proportional hazards model for the subdistribution of a competing risk. Publ Am Stat Assoc.

[27] Gray JR (1988). A class of K-sample tests for comparing the cumulative incidence of a competing risk. Ann Stat.

[28] Brock MV, Hooker CM, Ota-Machida E (2008). DNA methylation markers and early recurrence in stage I lung cancer. N Engl J Med.

[29] Koo HK, Jin SM, Lee CH (2011). Factors associated with recurrence in patients with curatively resected stage I-II lung cancer. Lung Cancer.

[30] Gu J, Lu C, Guo J (2013). Prognostic significance of the IASLC/ATS/ERS classification in Chinese patients-a single institution retrospective study of 292 lung adenocarcinoma. J Surg Oncol.

[31] Hung JJ, Yeh YC, Jeng WJ (2014). Predictive value of the International Association for the Study of Lung Cancer/American Thoracic Society/European Respiratory Society classification of lung adenocarcinoma in tumor recurrence and patient survival. J Clin Oncol.

[32] Yoshino I, Yohena T, Kitajima M (2001). Survival of non-small cell lung cancer patients with postoperative recurrence at distant organs. Ann Thorac Cardiovasc Surg.

[33] Hung JJ, Jeng WJ, Hsu WH (2012). Predictors of death, local recurrence, and distant metastasis in completely resected pathological stage-I non-small-cell lung cancer. J Thorac Oncol.

[34] Zhang Y, Zheng D, Xie J (2018). Development and validation of web-based nomograms to precisely predict conditional risk of site-specific recurrence for patients with completely resected non-small cell lung cancer: a multiinstitutional study. Chest.

[35] Maemondo M, Inoue A, Kobayashi K (2010). Gefitinib or chemotherapy for non-small-cell lung cancer with mutated EGFR. N Engl J Med.

[36] Mok TS, Wu YL, Thongprasert S (2009). Gefitinib or carboplatin-paclitaxel in pulmonary adenocarcinoma. N Engl J Med.

